# Association of virulence plasmid and antibiotic resistance determinants with chromosomal multilocus genotypes in Mexican *Salmonella enterica *serovar Typhimurium strains

**DOI:** 10.1186/1471-2180-9-131

**Published:** 2009-07-03

**Authors:** Magdalena Wiesner, Mussaret B Zaidi, Edmundo Calva, Marcos Fernández-Mora, Juan J Calva, Claudia Silva

**Affiliations:** 1 Departamento de Microbiología Molecular, Instituto de Biotecnología, Universidad Nacional Autónoma de México, Cuernavaca, México; 2 Laboratorio de Investigación, Hospital General O'Horan, Mérida, México; 3 Instituto Nacional de Ciencias Médicas y de la Nutrición "Salvador Zubirán", México City, México

## Abstract

**Background:**

Bacterial genomes are mosaic structures composed of genes present in every strain of the same species (core genome), and genes present in some but not all strains of a species (accessory genome). The aim of this study was to compare the genetic diversity of core and accessory genes of a *Salmonella enterica *subspecies *enterica *serovar Typhimurium (Typhimurium) population isolated from food-animal and human sources in four regions of Mexico. Multilocus sequence typing (MLST) and macrorestriction fingerprints by pulsed-field gel electrophoresis (PFGE) were used to address the core genetic variation, and genes involved in pathogenesis and antibiotic resistance were selected to evaluate the accessory genome.

**Results:**

We found a low genetic diversity for both housekeeping and accessory genes. Sequence type 19 (ST19) was supported as the founder genotype of STs 213, 302 and 429. We found a temporal pattern in which the derived ST213 is replacing the founder ST19 in the four geographic regions analyzed and a geographic trend in the number of resistance determinants. The distribution of the accessory genes was not random among chromosomal genotypes. We detected strong associations among the different accessory genes and the multilocus chromosomal genotypes (STs). First, the *Salmonella *virulence plasmid (pSTV) was found mostly in ST19 isolates. Second, the plasmid-borne betalactamase *cmy-2 *was found only in ST213 isolates. Third, the most abundant integron, IP-1 (*dfrA12*, *orfF *and *aadA2*), was found only in ST213 isolates. Fourth, the *Salmonella *genomic island (SGI1) was found mainly in a subgroup of ST19 isolates carrying pSTV. The mapping of accessory genes and multilocus genotypes on the dendrogram derived from macrorestiction fingerprints allowed the establishment of genetic subgroups within the population.

**Conclusion:**

Despite the low levels of genetic diversity of core and accessory genes, the non-random distribution of the accessory genes across chromosomal backgrounds allowed us to discover genetic subgroups within the population. This study provides information about the importance of the accessory genome in generating genetic variability within a bacterial population.

## Background

Bacterial genomes are mosaic structures composed of genes present in every strain of the same species (core genome), and genes present in some but not all isolates of a species (accessory genome) [1-3]. Genomic and population studies have shown that core and accessory genes often display distinct evolutionary histories, mainly due to the differential degree of mobility and selective pressures to which each category is subjected. It is accepted that the evolutionary histories of accessory genes are more complex than those of housekeeping genes [3,4]. Therefore, it is desirable to study core and accessory genes to better understand the population structure of a bacterial species [3,5].

*Salmonella enterica *is considered by population geneticists as the paradigm of a clonal bacterial species, that displays low levels of recombination and has mainly evolved by point mutations [6-8]. *Salmonella enterica *is subdivided in seven subspecies, the strains responsible for almost all the *Salmonella *infections in humans and warm-blooded animals belong to subspecies *enterica*. *Salmonella enterica *subspecies *enterica *has more than 1,500 described serovars [9]. To discriminate clones within serovars, macrorestriction analysis by pulsed-field electrophoresis (PFGE) and phage-typing are frequently used as subtyping techniques. More recently, multilocus sequence typing (MLST) has become an important tool for the study of *Salmonella *strains [10-13].

**Figure 1:**
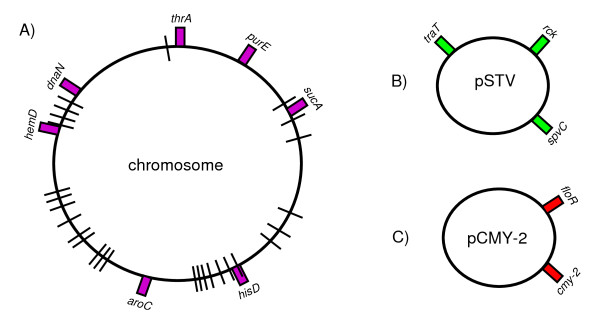
**Schematic representation of the molecular markers used to study core and plasmid accessory genes of Typhimurium from Mexico**. A) The chromosomal variation was addressed by multilocus sequence typing using partial sequences of the seven housekeeping genes [53], denoted by boxes on the chromosome of strain LT2 [GenBank:AE006468] [46], and by macrorestriction analysis using the rarely cutting enzyme *Xba*I resolved by pulsed-field electrophoresis, represented by lines crossing the chromosome at several points. B) The presence of the Typhimurium virulence plasmid (pSTV) [GenBank:AE006471] was determined by PCR amplification of three genes involved in virulence *spvC*, *rck *and *traT *[19,28], and by Southern hybridisation on plasmid profiles using *spvC *as probe. C) The presence of the plasmid-borne *cmy-2 *gene, conferring resistance to extended spectrum cephalosporins [GenBank:NC_011079] [30,31], was determined by PCR and by Southern hybridisation on plasmid profiles. The chloramphenicol determinant *floR *was also assessed, since it has been reported that both resistances are often encoded by the same plasmid [48].

**Figure 2:**
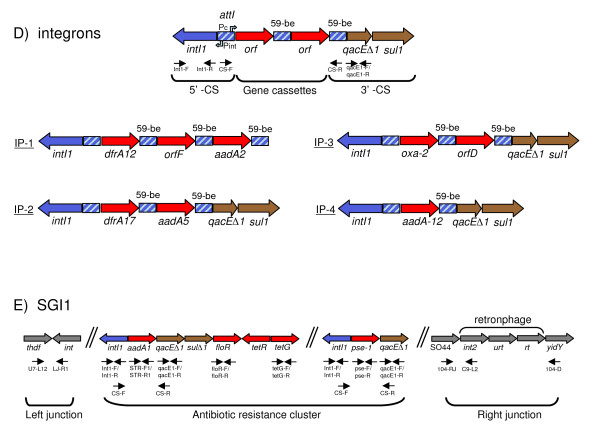
**Schematic representation of the molecular markers used to study the integrons of Typhimurium from Mexico**. A) Diagrammatic representation of the basic features of a class 1 integron [68]. The positions of the primers [see Additional file 3] used to amplify the different regions are shown by arrows. A class 1 integron consist of two conserved segments (5'-CS and 3'-CS) separated by a variable region that may contain an array of one or more gene cassettes. The 5'-CS includes the gene for the integrase (*intI1*), the promoters for the expression of the integrase (P_int_) and the gene cassettes (P_c_), and an adjacent *attI *recombination site, where the cassettes are integrated. Gene cassettes consist of a single promoter-less gene and a recombination site known as a 59-base element (59-be or *attC*), which is recognized by the site-specific recombinase (*intI1*). The 3'-CS includes *qacEΔ1 *and *sul1 *genes, determining resistance to quaternary ammonium compounds and to sulphonamide, respectively. The structure of the integron profiles found here, IP-1, IP-2, IP-3 and IP-4, are shown with their corresponding gene cassettes. B) Diagram of the regions of the *Salmonella *genome island 1 (SGI1) [43,44] that were studied. The positions of the primers [see Additional file 3] used to amplify the different regions are shown by arrows. The insertion of the island in the chromosome was detected by amplification of the right and left junctions; from the antibiotic resistance cluster the two integron-born gene cassettes (*aadA2 *and *pse-1*), *floR *and *tetG *were amplified.

*Salmonella enterica *subspecies *enterica *serovar Typhimurium (Typhimurium) is considered a broad host range serovar, usually associated with gastroenteritis in a broad range of phylogenetically unrelated host species [14-16]. The aim of this study was to compare the genetic diversity of core and accessory genes of a set of Typhimurium isolates sampled from food-animal and human sources in four geographic regions of Mexico. MLST and macrorestriction PFGE fingerprints were used to address the core genetic variation. To evaluate the distribution and genetic variation of the accessory genome, genes involved in pathogenesis and antibiotic resistance were selected. Schematic representations of the molecular markers assessed in this study are presented in Figures 1 and 2, and a brief description of them is presented below.

MLST is based on allelic differences in the nucleotide sequences of housekeeping genes among bacterial strains of a given species (Figure 1A) [5,17]. Macrorestriction analysis uses endonucleases that cut DNA at rare restriction sites, generating large fragments that are resolved by PFGE (Figure 1A). This methodology exhibits mostly chromosomal variation, but large plasmids can also be observed within the fingerprint [18].

For the accessory genome, we determined the presence of the Typhimurium virulence plasmid (pSTV). This plasmid has been extensively studied in regard to its role in invasiveness in the murine model [19-23]; its importance in human systemic infections is still controversial [24-27]. Three genetic markers were used to determine the presence of pSTV: *spvC*, *rck *and *traT*, that are genes involved in resistance to serum and survival in macrophages (Figure 1B) [19,28].

The antibiotic resistance determinants studied were those contained in integrons, and the presence of the plasmid-borne *cmy-2 *gene (Figure 1C), conferring resistance to extended spectrum cephalosporins. The *cmy-2 *gene is of major public health relevance since it confers resistance to ceftriaxone, the drug of choice for treatment of children with invasive *Salmonella *infections. In a previous study, we reported the rapid dissemination of this resistance in Typhimurium from Yucatán, Mexico, and its association with systemic infections in children [29]. Most *cmy-2 *genes have been located in large plasmids (> 100 kb), and were not found as an integron-born cassette [30,31].

The integron is a recombination and expression system that captures genes as part of a genetic element called a gene cassette (Figure 2A). Class 1 integrons are found extensively in clinical isolates, and most of the known antibiotic resistance gene cassettes belong to this class [32-35]. They are frequently located on plasmids and transposons, which further enhances the spread of the gene cassettes [32].

Class 1 integrons have been detected in different *Salmonella *serovars in many countries [36-41]. Among the most studied cases are the chromosomally located integrons present in the so-called *Salmonella *genomic island 1 (SGI1) (Figure 2B). SGI1 is a 43 kb integrative-mobilizable chromosomal element on which antibiotic resistance genes are clustered, flanked by two class 1 integrons [42,43]. The first cassette carries the *aadA2 *gene, which confers resistance to streptomycin and spectinomycin, and the second cassette contains *pse-1*, which confers resistance to ampicillin. In between them are *floR*, *tetR *and *tetG *genes, conferring resistance to chloramphenicol-florfenicol and tetracycline. A cryptic retronphage element is found as the last element of SGI1 in Typhimurium strains [43,44].

In the present work, analysis of the whole set of genetic markers targeting both housekeeping and accessory genes allowed us to determine genetic subgroups within the Mexican Typhimurium population.

## Results

### Distribution, genetic relatedness and antimicrobial resistance of MLST genotypes

The multilocus genotype for 114 Typhimurium isolates sampled from food-animal and human sources in four regions of Mexico, was determined. The seven-locus scheme recommended in the *Salmonella *MLST database [45] was applied to 66 isolates, in order to compare the diversity of our isolates with those reported in the database. The partial sequences of seven housekeeping genes revealed a low level of genetic variation; among the 3,336 nt only four substitutions were detected, yielding four multilocus genotypes or sequence types (ST) (Table 1). Thus, three novel alleles were identified: *purE*70, which consisted of a synonymous substitution, *purE*110, which contained one synonymous and one non-synonymous substitution, as compared with the *purE*5 allele present in most of the Typhimurium strains reported; and *sucA*144 which consisted of a synonymous substitution, as compared with the predominant *sucA*9 allele. ST19 is the predominant Typhimurium genotype in the MLST database (227 out of 391 Typhimurium entries) and has a worldwide distribution (24 countries, representing all continents). STs 213 and 429 have been reported only in Mexico, while ST302 has been reported in Mexico and Zimbabwe [45]. Despite the limitations of an analysis based on only four substitutions, an eBURST analysis of clonal relatedness among the different STs was consistent with the notion of ST19 as the founder genotype of the clonal complex, with the other three STs linked to ST19 as single-locus variants [see Additional file 1]. For the remaining 48 isolates we applied a three-gene scheme (see Methods) that allowed us to discriminate among STs (Table 1). The most abundant genotypes, ST213 and ST19, were found in the four geographic regions and in almost all the sampled years (Table 1). These genotypes presented a differential distribution among the sources of isolation (Table 2). Interestingly, ST213 was more prevalent in food-animals than in humans, where ST19 was predominant (59% vs 27%; p = 0.001, OR = 3.9).

**Table 1:** Allelic profiles and sequence types (STs) assigned in the *Salmonella *MLST database for the Mexican Typhimurium strains.

	Multilocus allelic profile^a^							No of isolates^b^				
				
ST	*aroC*	*dnaN*	*hemD*	*hisD*	*purE*	*sucA*	*thrA*	Seven^b^	Three^b^	Total	States^c^	Years
19	10	7	12	9	5	9	2	24	17	41	YU, MI, SL, SO	2000–2005
213^d^	10	7	12	9	70^d^	9	2	37	31	68	YU, MI, SL, SO	2001–2005
302^d^	10	7	12	9	110^d^	9	2	4	0	4	SO	2002–2004
429^d^	10	7	12	9	5	144^d^	2	1	0	1	MI	2003

^a ^Allele and ST numbers were those assigned in the *Salmonella *MLST database [45].

^b ^Number of strains analyzed using the seven-locus or the three-locus scheme (see methods for details).

^c ^YU, Yucatán; MI, Michoacán; SL, San Luis Potosí; SO, Sonora.

^d ^Novel alleles and sequence types (ST) obtained in this work study.

**Table 2:** Distribution of human and animal strains of STs 19 and 213 harbouring pSTV or pCMY-2.

	Number of strains (%)			
	
Source	ST19	ST213	pSTV	pCMY-2
Human	30 (73)	28 (41)	25 (76)	23 (64)
Animal	11 (27)	40 (59)	8 (24)	13 (36)

Total	41	68	33	36

**Figure 3:**
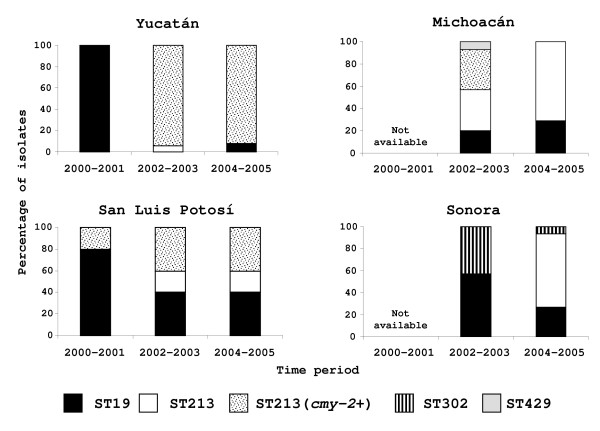
**Distribution of the percentage of Typhimurium STs according to the time period and geographic location**.

We found a temporal pattern in which the derived ST213 is replacing the founder ST19 in the four geographic regions (Figure 3). ST19 was predominant in Yucatán and San Luis Potosí in the first period (2000–2001). During the second period (2002–2003), ST213 was the most abundant genotype in Yucatán, Michoacán and San Luis Potosí; only in Sonora ST19 was the most abundant genotype. However, by the end of the time period studied (2004–2005), ST213 was the predominant genotype in all four states (Figure 3).

We found a strong association between STs and antimicrobial resistance. ST213 isolates presented higher percentages of resistance (> 50%) than ST19 isolates, the only exception was ciprofloxacin for which all the isolates were susceptible (Table 3). All the isolates resistant to ceftriaxone belonged to ST213, while all the isolates from STs 19, 302 and 429 were ceftriaxone susceptible. The group of isolates resistant to ceftriaxone (n = 36) was associated with very high percentages (> 95%) of resistance to ampicillin, chloramphenicol, sulfisoxazole, streptomycin and tetracycline, here after referred to as the pentaresistant phenotype.

**Table 3:** Percentage of antimicrobial resistant strains for the two main Typhimurium STs.

	Antimicrobial resistance										
	
	AMP^a^	CHL	SSS	STR	TET	GM	KM	NAL	SXT	CIP^b^	CRO
ST19	61	51	75	80	75	7	10	10	22	0	0
ST213(*cmy-2*)^c^	68 (97)	90 (94)	98 (97)	97 (97)	97 (100)	59 (55)	37 (33)	72 (61)	82 (92)	0	53 (100)

^a ^AMP:ampicillin, CHL: chloramphenicol, SSS: sulfisoxazole, STR: streptomycin, TET: tetracycline, GM: gentamicin, KM: kanamycin, NAL: nalidixic acid, SXT: timethoprim-sulfametoxazole, CIP: ciprofloxacin, CRO: ceftriaxone.

^b ^All the strain were sensitive to CIP according with CLSI [78], including twelve strains with low-level resistance [see Additional file 2].

^c ^The number in parenthesis is the percentage corresponding to ST213 strains positive for *cmy-2*.

The resistance patterns varied across geographic locations. Yucatán was the state with the higher level of multidrug resistance, with an average of seven resistances per isolate; while Sonora presented the lowest levels of resistance with an average of four. Michoacán and San Luis presented intermediate values, both with an average of six. Furthermore, the ST213 ceftriaxone resistant isolates displayed a differential geographic pattern, ranging from 97% of the ST213 isolates in Yucatán to 0% in Sonora, with intermediate levels in Michoacán and San Luis Potosí (Figure 3).

### Distribution and associations of pCMY-2

Isolates resistant to ceftriaxone were subjected to PCR analysis to detect the presence of the *bla*_CMY-2 _gene (Figure 1C). All 36 isolates resistant to ceftriaxone were positive, whereas the 12 sensitive isolates tested were negative [see Additional file 2]. Sequencing (564 bp) of *cmy-2 *for 16 isolates revealed that all carried an identical allele, suggesting a common origin. The BLAST searches showed that this allele was identical to most of the 100 hits targeting the Enterobacteriaceae (*Escherichia*, *Salmonella*, *Klebsiella*, *Proteus *and *Citrobacter*).

To determine the location of the *cmy-2 *gene, plasmid profiles for 25 isolates were hybridized with the corresponding radioactive probe. In all the isolates positive for *cmy-2 *the probe hybridized with a plasmid of about 200 kb, hereafter referred to as pCMY-2; while the negative isolates did not yield a signal. The strength of the association between pCMY-2 and chromosomal genotype was confirmed (p = 0.001, OR = 93), since all the isolates harbouring pCMY-2 were ST213 (Table 3 and Additional file 2).

### Distribution, genetic diversity and associations of pSTV

The presence of pSTV was first assessed by PCR amplification of *spvC*. Only 30% of the isolates were positive for *spvC *[see Additional file 2]. To confirm the presence or absence of the pSTV we amplified *rck *and *traT *for all 33 *spvC *positive isolates, and for 19 *spvC *negative isolates. All *spvC *positive isolates amplified *traT *and *rck*, with the exception of two isolates that did not amplify *rck *(slhs02–20 and slres03–40; see Additional file 2); while the *spvC *negative isolates did not produce amplifications with either *rck *or *traT*.

To evaluate the genetic diversity of pSTV we determined the nucleotide sequences of *spvC *for 16 representative isolates [see Additional file 2]. All *spvC *sequences (513 bp) were identical to each other, displaying only one nucleotide substitution with respect to the sequence of strain LT2 [GenBank:AE006471] [46]. We further determined the sequences of *traT *and *rck *for 11 and 9 isolates, respectively. The *traT *(450 bp) and *rck *(429 bp) sequences were also identical to each other and to the sequence of strain LT2. These results show pSTV with a low level of genetic diversity distributed in the four geographic regions and recovered during the five sampled years.

We confirmed the presence of pSTV and determined its approximate size by Southern blot hybridization of plasmid profiles for 10 isolates. All the isolates that where positive for the amplification of *spvC*, *rck *and *traT *hybridized with a plasmid of the same size of that of the pSTV of strain LT2 (about 94 kb) [46], and all the negative controls produced no signal with the *spvC *probe. However, one of the isolates that did not amplify *rck *hybridized with a larger plasmid of about 120 kb, indicating that this pSTV is different, probably due to the insertion of mobile elements, such as transposons, as previously reported [19,47].

**Figure 4:**
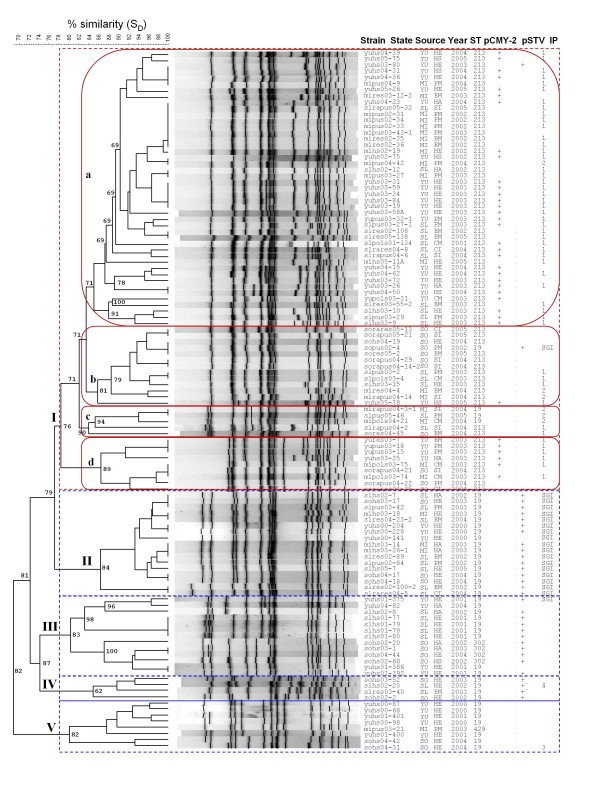
**Dendrogram depicting the relationships of Mexican Typhimurium strains based on *Xba*I restriction patterns resolved by PFGE**. The fingerprints were clustered by the UPGMA algorithm using Dice coefficients with 1.5% band position tolerance. Detailed information about strains can be found in Additional file 2. The strain column depicts the nomenclature used in the MLST database for the MEXSALM collection. Abbreviations for the state column: YU, Yucatán; MI, Michoacán; SL, San Luis Potosí; SO, Sonora. Abbreviations for the source column: HE, human enteric; HS, human systemic; HA: human asymptomatic; PM, pork meat; SI, swine intestine; BM, beef meat; CM, chicken meat; BI, beef intestine. The strains positive for the presence of pCMY-2 or pSTV are indicated by a plus symbol (+), the two strains marked with a +' in the pSTV column are the strains for which *rck *could not be amplified. The nomenclature of integron profiles (IP1–IP4) is explained in the text. The five main clusters (I-V) are highlighted by dotted rectangles, and the four subgroups (a, b, c and d) in cluster I are indicated by oval boxes. Cophenetic values are shown for the clusters formed above 90% similarity.

pSTV was present in 29 ST19 isolates (68%), the four ST302 isolates (100%) and only one ST213 isolate (1%; yuhs03–80; Figure 4 and Additional file 2). This finding indicates that pSTV was not randomly distributed among isolates, since 60% of the isolates were ST213, and showed a significant association between ST19, and pSTV (p = 0.001, OR = 144). Human isolates harboured pSTV significantly more than food-animal isolates (43% vs. 16%, p = 0.002, OR = 4.1), demonstrating a significant association with the human host. Many of these isolates were isolated from humans with diarrhea or asymptomatic infection; only one of the six isolates from systemic infections had pSTV [see Additional file 2], indicating that its presence does not necessarily cause extra-intestinal infections.

### Detection and associations of integrons

All 114 isolates were assessed for the presence of integrons using primers targeting the CS regions (Figure 2 and Additional file 3), which amplify the cassettes inserted in integrons. A high proportion (66%) of the isolates produced an amplification product [see Additional file 2]. The most abundant one (42% of the isolates) was of about 2,000 bp, and was designated as integron profile 1 (IP-1). The nucleotide sequence of this integron for 12 isolates showed that it was composed of an array of three cassettes containing the genes *dfrA12*, *orfF *and *aadA2 *(Figure 2A). The sequences (1,816 bp) were almost identical to each other (only one substitution) and to most of the sequences retrieved after BLAST searches from GenBank (see details in the Discussion section). An integron of about 1,650 bp was present in six isolates and designated as integron profile 2 (IP-2) (Figure 2A). Nucleotide sequencing showed that it was composed of two cassettes containing the genes *dfrA17 *and *aadA5*. The sequences (1,573 bp) of the six isolates were identical to each other and to most of the GenBank sequences (see details in the Discussion section). Two isolates produced amplification bands of about 1,300 and 1,000 bp; sequence determination showed that they harboured *oxa-2 *and *orfD*, and *aadA12 *cassettes, and were designated as IP-3 and IP-4, respectively (Figure 2A and Additional file 2). BLAST searches showed that the sequence of IP-3 (*oxa-2 *and *orfD*) was identical to an integron of *Aeromonas hydrophila *from Taiwan [GenBank:DQ519078], and the sequence of IP-4 (*aadA12*) was identical to an integron of *Yersinia enterocolitica *from Spain [GenBank:AY940491] (Figure 2A).

The second most abundant integron profile (16% of the isolates) was conformed by two amplification bands of about 1,000 and 1,200 bp. This is typically the profile recovered from the SGI1, and therefore was designated as IP-SGI1 (Figure 2B and Additional file 2). Sequence determination for three isolates showed that the 1,000 bp cassette contained *aadA2 *and that the 1,200 bp cassette coded for *pse-1*, which are the most commonly found integrons in the SGI1. All the isolates were positive for the amplification of *pse-1*and *aadA2 *using primers specific for these genes (Figure 2B and Additional file 3). To confirm the insertion of the complete SGI1 in the chromosome, we performed PCR assays to amplify the left and right junctions. All the isolates (n = 19) harbouring the IP-SGI amplified the left junction, the right junction, and were positive for the amplification of the cryptic retronphage on the right junction [see Additional file 2]. Isolates harbouring other integrons did not amplify any of the junctions of the SGI1. To further characterize the SGI1, we amplified the *tetG *and *floR *genes that are in between the two integrons. Only the isolates harbouring the IP-SGI1 produced strong amplification products with *tetG*, and all were positive for *floR*; however, other chloramfenicol resistant isolates also amplified *floR*. All the *cmy-2 *positive isolates (n = 36) were positive for *floR*, which is in agreement with the report by Doublet et al. (2004) that both resistances are often found in the same plasmid [11,48]. Thus, most of the *floR *positive isolates harboured SGI1 or pCMY-2, however, other chloramfenicol resistant isolates were positive for *floR*. Some of the isolates harbouring IP-2 showed weak amplification bands with *tetG *or *floR *primers, probably due to the presence of related but divergent genes conferring resistance to tetracycline and chloramfenicol [see Additional file 2]. Two significant associations among integrons and the other molecular markers are worthy of mention. First, all IP-1 were carried by ST213 isolates (p = 0.001, OR = 211), either *cmy-2 *positive or negative. Second, all the isolates with SGI1 were ST19 and carried pSTV (p = 0.001, OR = 119), the only exception was one isolate that did not carry pSTV (yuhs00–141; Figure 4 and Additional file 2).

To determine the location of the integrons, we performed Southern hybridization experiments using fragments of the *intI1 *and *aadA2 *genes as probes on the plasmid profiles of eight representative isolates. Three of the five isolates harboring IP-1 hybridized with a plasmid of about 100 kb, the remaining two IP-1 isolates hybridized with a plasmid of about 150 kb. The isolate harboring IP-2 hybridized with a plasmid of about 150 kb, IP-3 with a plasmid of about 35 kb, and IP-4 with a plasmid of about 100 kb.

### Detection of *intI1 *and *qacEΔ1*

To further characterize the 5' and 3' CSs of integrons we amplified *intI1 *and *qacEΔ1 *(Figure 2A). All isolates displaying IP-2, IP-SGI1, IP-3 and IP-4 showed strong amplification bands for *intI1 *and *qacEΔ1*, which indicates that they have 5' and 3' CSs typical of class1 integrons. All the isolates with IP-1 amplified a strong band with *intI1*, but only four isolates amplified strong bands for *qacEΔ1*. Most of the isolates with IP-1 (76%) did not amplify *qacEΔ1 *or produced very weak bands (16%) [see Additional file 2]. This result suggests that most of these integrons contain an unusual 3' CS, as recently reported for this integron in *Salmonella *and *Staphylococcus *[40,49-51]. Twenty isolates that did not amplify the cassette region using the CS-F and CS-R primers were selected to test the amplification of *intI1 *and *qacEΔ1*. Most of these isolates did not produce amplifications, or produced very weak bands; only four isolates presented an intense *intI1 *band.

### Macro-restriction PFGE dendrogram and association among molecular markers

The PFGE fingerprints were clustered using the UPGMA algorithm. The dendrogram was divided in five clusters using a cut-off value of 78% similarity (Figure 4). Cluster I grouped all the ST213 isolates and four ST19 isolates. Using the information provided by the accessory genes, this cluster can be further subdivided in four main groups. Group Ia contained only ST213 isolates from three different states, many of which carried *cmy-2 *and IP-1. Groups Ib and Ic contained ST213 isolates mostly without *cmy-2 *and ST19 isolates without pSTV, and comprising five of the six IP-2. Group Id was similar to group Ia; it contained ST213 isolates, most of which harboured *cmy-2 *and IP-1. It is distinguished from groups Ia and Ib by the lack of a large restriction fragment of about 665 kb. Cluster II was formed by ST19 isolates carrying both pSTV and SGI1. Clusters III and IV grouped ST19 isolates and the four ST302 strains, most of them carrying pSTV. Cluster IV contained the two ST19 isolates for which *rck *could not be amplified, and one of them carried the IP-4 integron. Finally, cluster V was composed by ST19 strains lacking pSTV. A few exceptions to these general patterns were detected, such as a cluster I ST213 isolate harbouring pSTV (yuhs03–80) or a ST19 isolate harbouring pSTV and SGI1 in cluster I (sorapus02–4). The whole set of genetic markers targeting both housekeeping and accessory genes allowed us to discover genetic subgroups within the isolate set.

## Discussion

### Low genetic diversity of core and accessory genes

Both housekeeping and accessory genes displayed extremely low levels of genetic diversity; even the third codon positions were invariable. The low genetic diversity and the clonal pattern of descent of accessory elements could be explained by several evolutionary processes, such as rapid clonal expansion of the population, genetic drift, the existence of barriers to genetic exchange among subgroups within the population, or a combination of these possibilities [4,5,8,52,53]. Most of the genetic diversity was provided by the presence/absence of accessory genes, including plasmids (pSTV and pCMY-2), integrons (IP-1 to 4 and IP-SGI), and a chromosomally inserted island (SGI1), rather than by nucleotide polymorphisms. This result is in agreement with the conclusions derived from *Salmonella *whole genome comparisons and microarray data [53-56].

### Geographic distribution of multilocus genotypes and antimicrobial resistance

Both MLST and PFGE analysis revealed the presence of widely distributed Typhimurium clones that were isolated from human and food-animal sources, during different years and from diverse geographic locations in Mexico. Taken together, our results indicate that: 1) there are effective mechanisms for the dissemination of *Salmonella *throughout the country and, thus, the entire sample can be considered a single population; 2) the isolates found in food-animals and humans are related; and 3) the clones causing disease in humans do not differ from those circulating in healthy humans or animals. The observation that isolates from human and food-animal sources come from the same genetic pool is in agreement with our previous reports [29,57], and with studies from other parts of the world [10,13], supporting the hypothesis of *Salmonella *transmission through the food chain. The fact that the isolates causing disease (enteric or invasive) in humans are not distinct clones from those carried by healthy humans and animals, suggest differences in the bacterial inoculum, immune status of the host and modes of transmission. Furthermore, there may be differences in virulence determinants affecting the pathogenic capabilities, that cannot be distinguished by the methodologies applied in this study.

We found that the derived ST213 is replacing the founder ST19. Genotype replacement has been previously reported for *Salmonella*, as well as other bacterial species and virus. For example, the replacement of Typhimurium DT204 by the globally disseminated DT104 has been reviewed elsewhere [58,59]. The comparison of historic (1988–1995) and contemporary (1999–2001) serovar Newport isolates showed that they belonged to clearly separated PFGE clusters [60]. Shifts in the clonal prevalence of methicillin-resistant *Staphylococcus aureus *have been documented in hospitals from Spain and Portugal [61,62]. These results show that shorts periods of time are enough to observe drastic changes in genotype circulation, as reported in the present study. The geographic differences in the number of resistance determinants in ST213, in particular, the extended-spectrum cephalosporin resistance in isolates from Yucatán (97%) as compared with isolates from Sonora (0%), could be reflecting regional differences in the use of antibiotics in animal production. In this study we found strong associations among antimicrobial determinants. For example, all the *cmy-2 *positive isolates carried IP-1, were positive for *floR *and presented the pentaresistant phenotype. This finding is in line with several studies that report the spread of large transferable plasmids carrying multiple resistance determinants [18,28-30,34,36,48,63,64]. The pentaresistant phenotype was also displayed by isolates harbouring the chromosomally inserted SGI1, which demonstrates that the same resistance phenotype can have a completely different genetic background, as reported by others [18,65].

Because of the recent dissemination of *cmy-2 *positive Typhimurium isolates in Mexico [29], the genotypic characterization of our isolates is of public health relevance and provides useful information that can be used to improve the integrated food chain surveillance system that is being established in this developing country [57].

### Distribution of pSTV among hosts and chromosomal genotypes

Whether the pSTV is necessary to produce systemic infections in humans has been subject of intense debate. Some authors claim that there is lack of evidence of an association between the carriage of pSTV and human bacteremia [24]. Other authors suggest that *spv *genes promote the dissemination of Typhimurium from the intestine [26]. In a recent report, Heithoff et al. (2008) found that all the Typhimurium strains isolated from humans with bacteremia or animals possessed pSTV, while 34% of the strains isolated from human gastroenteritis lacked pSTV [66]. These results are in contrast with the data obtained in the present study. Unexpectedly, we found that less than half of all human strains harboured pSTV, and only one of the six isolates recovered from patients with systemic infection had pSTV, supporting the view that pSTV is not essential for human systemic infections. On the other hand, pSTV was significantly associated with human isolates (Table 2), indicating that the ST19-pSTV genotypes are adapted to the human host, while ST213 genotypes are adapted to both animal and human hosts. In conclusion, our data supports the notion that pSTV has a role in host adaptation [14], however, are not consistent with the view that pSTV is associated with systemic infection in humans.

There are some reports describing the differential distribution of pSTV within Typhimurium genotypes. Olsen et al. (2004) performed plasmid transfer experiments with the aim of demonstrating that different Typhimurium genotypes differed in their ability to obtain and express pSTV [21]. Ou and Baron (1991) observed that the introduction of a plasmid from a highly virulent strain did not increase virulence in all strains, particularly in those that were moderately virulent with their own plasmids, or did not contain a pSTV [22]. These reports highlight the importance of the genomic background in the interaction with the pSTV. In the present study we found a statistical association between genomic background and the presence of pSTV. This finding is also consistent with the PFGE dendrogram, in which subgroups are strongly associated with the presence or absence of pSTV.

We found that almost all the isolates harbouring the pSTV were ST19 (85%), while all the isolates harbouring pCMY-2 were ST213. Since ST213 is a recently derived genotype from ST19, and ST213 isolates did not harbour pSTV, it is appealing to speculate that ST213 arose as a derived clone lacking pSTV (which is a idiosyncratic plasmid of *Salmonella*), and that this condition allowed the acquisition of pCMY-2 (which is a broad host range plasmid of Enterobacteriaceae).

### Distribution of pCMY-2 among chromosomal genotypes

Since the presence of pCMY-2 in *Salmonella *is very recent compared to other Enterobacteriaceae, its differential distribution within genotypes of a single *Salmonella *serovar is scarcely documented. The association of the AmpC phenotype with a subgroup of genotypes has been documented mainly for Newport. Gupta et al. (2003) found that the isolates with this phenotype presented highly related PFGE restriction patterns that differed from those of the susceptible isolates [63]. Harbottle et al. (2006) found that all the Newport isolates with the multidrug resistant AmpC phenotype were grouped in a single PFGE cluster, and belonged to only two of the 12 STs present in the sample [13]. Zhao et al. (2007) found that the cephalosporin resistant Newport isolates presented related PFGE fingerprints and differed from those of susceptible isolates. Similar findings were reported for serovar Dublin [41]. On the other hand, Alcaine et al. (2005) studied Typhimurium, Agona and Schwarzengrund isolates from dairy farms, and did not find particular STs associated with the presence of *cmy-2*, concluding that *cmy-2 *positive isolates evolved independently by horizontal gene transfer [11]. Our data strongly suggest that in the Mexican Typhimurium population pCMY-2 is associated with multidrug resistance and is harboured only by ST213 genotypes.

### Integrons as source of strain diversity

In this work we found four types of integrons encompassing nine different genes (*aadA2*, *aadA5*, *aadA12*, *dfrA12*, *dfrA17*, *oxa-2*, *pse-1*, *orfD*, and *orfF*). Seven of them were genes encoding antimicrobial resistance determinants well known to be associated with integrons in the Enterobactariaceae [32,67], and two were open reading frames with unknown function but also previously reported as gene cassettes [32]. To a large extent, the presence of integrons and plasmids defined the distinctive features of the main genetic subgroups, and provided strain diversity to an otherwise almost uniform population. These elements are known to be an integral part of the mobile or floating genome, and represent a fundamental resource for bacterial evolution [68-70].

The two integrons designated in this study as IP-1 and IP-2 have been found in several *Salmonella *serovars (e. g. Anatum, Branderup, Brikama, Enteritidis, Mbandaka, Rissen, Saintpaul and Typhimurium), and in other Enterobacteriaceae, such as *E. coli *[37-41]. In a recent study these integrons were detected in three *Staphylococcus *species isolated in China [51], providing evidence of the successful spread of this integrons around the world and across bacterial phyla. BLAST searches showed the presence of the *dfrA12*, *orfF *and *aadA2 *integron in 47 isolates proceeding from Proteobacteria (Enterobacteriales, Pseudomonadales, Aeromonadales and Vibrionales) and Firmicutes (Bacillales and Lactobacillales). The majority of the nucleotide sequences from these isolates were identical, suggesting that this integron has been recently acquired by a broad range of bacterial species. In many of these cases the location of the integron in plasmids has been documented, in agreement with the results found in the present study, which may account for its widespread distribution.

In contrast to prior evidence of horizontal transfer of *dfrA12*, *orfF *and *aadA2 *across bacterial lineages, in the present study we found that the distribution of this integron was not random across chromosomal backgrounds, since these were found only in ST213 isolates. A similar situation was observed for SGI1, for which a rather narrow distribution was observed (mainly cluster II isolates), despite the proved mobility of SGI1 [42]. Our results provide evidence for the clonal dissemination of the island rather than lateral transfer among diverse genotypes. The association of pSTV with isolates harbouring SGI1 has been previously described [71,72]. Taken together, these results point out that although this Mexican Typhimurium population is exposed to a broad genetic pool of accessory genes, there are associations and restrictions among genomic backgrounds and the environmental floating genome.

## Conclusion

The analysis of core and accessory genes in Mexican Typhimurium isolates allowed us to identify genetic subgroups within the population. We found strong statistical associations among chromosomal genotypes and accessory genes. The general patterns of association can be summarized as follows: 1) the isolates harbouring pSTV were ST19 or ST302, 2) all the isolates with SGI1 were ST19 and most carried pSTV, 3) all the isolates harbouring pCMY-2 were ST213, and 4) all IP-1 were carried by ST213 isolates. The low genetic diversity and the clonal pattern of descent of accessory elements could be explained by a combination of evolutionary processes. This study provides information about the importance of the accessory genome in generating genetic variability within a bacterial population.

## Methods

### *Salmonella *isolates and antimicrobial susceptibility testing

This study used 114 Typhimurium isolates collected for a Mexican surveillance network comprised by four states. The geographic locations of these states range from the southeastern to the northwestern part of Mexico. The more distant states (Yucatán and Sonora) are about 2,000 km apart and the closest states (Michoacán and San Luis Potosí), about 450 km apart.

In all states, food-animal production is a major economic activity, and most of the circulating retail meat is locally produced. The sampling scheme was designed to follow the food chain in a temporal fashion; details about the epidemiologic design can be found in Zaidi et al. (2008). Briefly, isolates from ill humans were obtained from patients at state referral hospitals; isolates from asymptomatic humans were collected from the feces of kindergarten children; raw retail pork, beef and chicken were purchased at supermarkets, butcher shops and open markets; and intestines were obtained from food-animals at slaughter from municipal abattoirs [57,73]. The internal review boards and ethics committees of all collaborating hospitals in the surveillance network approved the protocol, and written informed consent was collected from the guardians of all participants to obtain fecal and/or blood samples, and use the clinical and microbiologic information for scientific studies [57].

We did not use a systematic randomization method for selecting strains for this study. Using a chart with a list of each isolate by city of origin, strains were manually selected by including at least one strain from animals, meat or humans from a total of 61 cities. The sample included 38 isolates from Yucatán, 22 from Michoacán, 32 from San Luis Potosí and 22 from Sonora. Sixty-two isolates were from human samples (45 with diarrhea, 11 asymptomatic and 6 with systemic infection), and 52 from food-animals (18 from pork, 14 from beef, 6 from chicken meat, 10 from swine intestine, and 4 from cattle intestine). Isolates collected during 2000 and 2001 were only available from Yucatán and San Luis Potosí. Isolates collected from 2002 to 2005 were available for all four states (Table 1 and Figure 3).

Isolates biochemically confirmed to be *Salmonella *were serotyped according to the Kauffmann-White scheme with commercial antisera, as described elsewhere [73]. All isolates were tested with the disk diffusion method [74] for susceptibility to ampicillin, chloramphenicol, sulfisoxazole, streptomycin, tetracycline, gentamicin, kanamycin, nalidixic acid, trimethoprim-sulfamethoxazole, ciprofloxacin and ceftriaxone. The minimum inhibitory concentrations (MICs) for ciprofloxacin and ceftriaxone were determined by agar dilution according to Clinical and Laboratory Standards Institute guidelines [75]. For the interpretation of MIC results for ciprofloxacin, high-level resistance was defined as a MIC value ≥ 2 μg/mL; low-level resistance was defined as a MIC value ≥ 0.25 μg/mL and ≥ 1 μg/mL.

### MLST analysis

Genomic DNA was extracted using the AquaPure Genomic DNA Kits (Bio-Rad Laboratories, Hercules, California, USA). PCR amplifications were performed with *Taq *DNA Polymerase (Invitrogen, Brazil), products were purified with a PCR purification kit from Qiagen (Valencia, California, USA) according to the manufacturer's recommendation, and submitted for sequencing at Macrogen (Seoul, South Korea).

MLST was based on the partial sequences (~450 bp) of the following seven housekeeping genes: *aroC, dnaN, hemD, hisD, purE, sucA *and *thrA*, according to the *Salmonella *MLST database [45]. The primers for PCR and sequencing were previously described by Kidgell et al. (2002) [53]. Sequences were edited and aligned using Clustal W as implemented in BioEdit [76], and submitted to the MLST website for allele number assignment. The different sequences at each locus were assigned to an existing or novel allele, and each unique allelic profile (or multilocus genotype) was assigned to a sequence type (ST).

The clonal relatedness of the STs was determined using eBURSTv3 [77]. This program discerns the most parsimonious patterns of descent of isolates within a clonal complex from the predicted founder. The primary founder is predicted on the basis of parsimony, as the ST that has the largest number of single-locus variants in the group or clonal complex. Clonal complexes are thought to emerge from the rise in frequency and subsequent radial diversification of clonal founders [77].

The MLST analysis for the first 66 isolates analyzed showed that mostly *purE *presented polymorphisms among the seven genes assessed. Since this gene had the ability to discriminate the three main STs present in the isolate set, we decided to implement an economical three-gene MLST for the remaining 48 isolates of the sample, as suggested elsewhere [10-12]. The genes selected were *purE*, *thrA *and *sucA*; the latter two on the basis of their variability among the *Salmonella *[45]. Only the seven-gene MLST data were submitted to the *Salmonella *MLST database.

### PFGE macro-restriction analysis

PFGE fingerprints for the isolates collected from 2002 to 2005 were previously generated for the surveillance network reported by Zaidi et al. (2008) [57]. For isolates collected during 2000 and 2001, the macro-restriction analysis was performed using the same conditions, following the methodology developed by the Centers for Disease Control and Prevention (USA) [78]. The *Xba*I restriction patterns were clustered using the unweighted pair-group method with arithmetic averages. The analyses were done with GelComparII using band matching and Dice coefficients with a 1.5% band position tolerance. The consistency of the PFGE clusters was obtained by calculating cophenetic values as implemented in GelComparII. This method calculates the correlation between the dendrogram-derived similarities and the matrix similarities.

### Detection of pSTV and pCMY-2

Additional file 3, lists the primers and conditions for detection of pSTV by PCR amplification of *spvC*, *rck *and *traT*, and the presence of *cmy-2*. To determine the size of pSTV and pCMY-2, plasmid profiles were generated by a modification of the alkaline lysis procedure [79]. The plasmid profile gels were transferred to positively charged membranes (Amersham Hybond™-N^+^) and hybridised with *spvC *and *cmy-2 *probes. Probes were derived from the PCR products and labelled radioactively with ^32^P. Hybridizations were performed under high stringency conditions at 65–68°C.

### Detection of integrons and SGI1

The primers and conditions used to detect integrons and SGI1 are listed in Additional file 3. Integrons were detected using primers CS-F and CS-R, targeting the 5' and 3' CS, which amplify the inserted cassettes (Figure 2). These primers were also used for integron sequence determination. For sequencing of IP-1, which contains three gene cassettes (*dfrA12*, *orfF *and *aadA2*), a third internal primer (STR-R1) targeting the region *aadA2 *was used. The isolates displaying the two integrons typical of SGI1 were subject to amplification of the left, right and retronphage junctions, as well as for the antimicrobial resistance genes *tetG*, *floR*, *pse-1 *and *aadA2*. To further characterize the 5' and 3' CS regions of integrons, as well as to search for isolates containing integrons without gene cassettes, the class 1 integrase (*intI1*) and *qacEΔ1 *genes were amplified.

To determine the location of integrons for some representative isolates, plasmid profiles were generated and transferred to positively charged membranes. Probes were derived from the PCR products of *intI1 *and *aadA2 *genes, and labelled radioactively with ^32^P. Hybridizations were performed under high stringency conditions at 68°C.

### Statistical Analysis

Statistical testing of differences in proportions was conducted using the chi-square test with Yates' correction; *p *values < 0.05 were considered significant. Strength of association between nominal variables was assessed by calculating the odds ratio (OR).

### Nucleotide accession numbers and database searches

Only one representative sequence for each of the alleles found was submitted to the GenBank database. The *spvC*, *rck*, *traT*, *aadA2 *and *pse-1 *partial sequences for strain sopus02–4 were submitted under accession numbers [GenBank:FJ460230], [GenBank:FJ460231], [GenBank: FJ460232], [GenBank:FJ460233] and [GenBank:FJ460234], respectively. The *cmy-2 *and IP-1 (*dfrA12*, *orfF *and *aadA2*) partial sequences of strain yuhs04–31 were submitted under accession numbers [GenBank:FJ460235] and [GenBank:FJ460236], respectively. IP-1 from strain sores04–45 was submitted under accession number [GenBank:FJ460237]. IP-2 (*dfrA17 *and *aadA5*) partial sequence from strain mirapus04-3-1 was submitted under accession number [GenBank:FJ460238]. IP-3 (*oxa-2 *and *orfD*) from strain sohs04–31 was submitted under accession number [GenBank:FJ460239]. IP-4 (*aadA12*) from strain slhs02–20 was submitted under accession number [GenBank:FJ460240]. The nucleotide sequences generated in this work were compared to public databases using the BLAST algorithm at NCBI [80].

## Authors' contributions

MW performed most of the MLST and part of the PFGE data, helped in the generation and analysis of the data from the accessory genes, and helped to draft the manuscript. MBZ provided the isolates, performed the antimicrobial susceptibility tests and most of the PFGE data, participated in the study design, performed the statistical analysis and helped to draft the manuscript. EC started the conception of the study, participated in its design and coordination, and helped to draft the manuscript. MFM participated in the performance of the laboratory work, such as the PCR assays, plasmid extraction procedures and southern hybridizations. JJC participated in the initial design of the epidemiological study and in the conception of this study. CS conceived and performed most of the work on the analysis of the accessory genome, helped in the generation of the MLST data, and drafted the manuscript. All authors read and approved the final manuscript.

## Supplementary Material

Additional file 1 **Figure S1 – Clonal complex for the four multilocus genotypes found in the Mexican Typhimurium population. Representation of the clonal relatedness of STs. Figure S1 – Clonal complex for the four multilocus genotypes found in the Mexican Typhimurium population**. The eBURST diagram show the genetic relationships for 66 Typhimurium strains based on the MLST data. ST 19 was unambiguously (100% bootstrap support) predicted as the founder genotype, with STs 213, 302 and 429 related as single locus variants of ST19. The size of the circles is proportional to the number of isolates belonging to each ST. Click here for file

Additional file 2 **Table S1 – Complete list of strains and results. The complete list of strains, sampling information and results of the genotypic and phenotypic characterization is presented. Table S1 – Complete list of strains and results**. The complete list of strains, sampling information and results of the genotypic and phenotypic characterization is presented. Click here for file

Additional file 3 **Table S2 – Primers used in this study. The primer sequences, amplification sizes, annealing temperatures and references are listed. Table S2 – Primers used in this study**. The primer sequences, amplification sizes, annealing temperatures and references are listed. Click here for file
