# Supplementary figures and images for: Association of virulence plasmid and antibiotic resistance determinants with chromosomal multilocus genotypes in Mexican Salmonella enterica serovar Typhimurium strains
Source: BMC Microbiol. 2009 Jul 3;9:131. doi: 10.1186/1471-2180-9-131 (PMC2715408; doi:10.1186/1471-2180-9-131)

## Slide 1
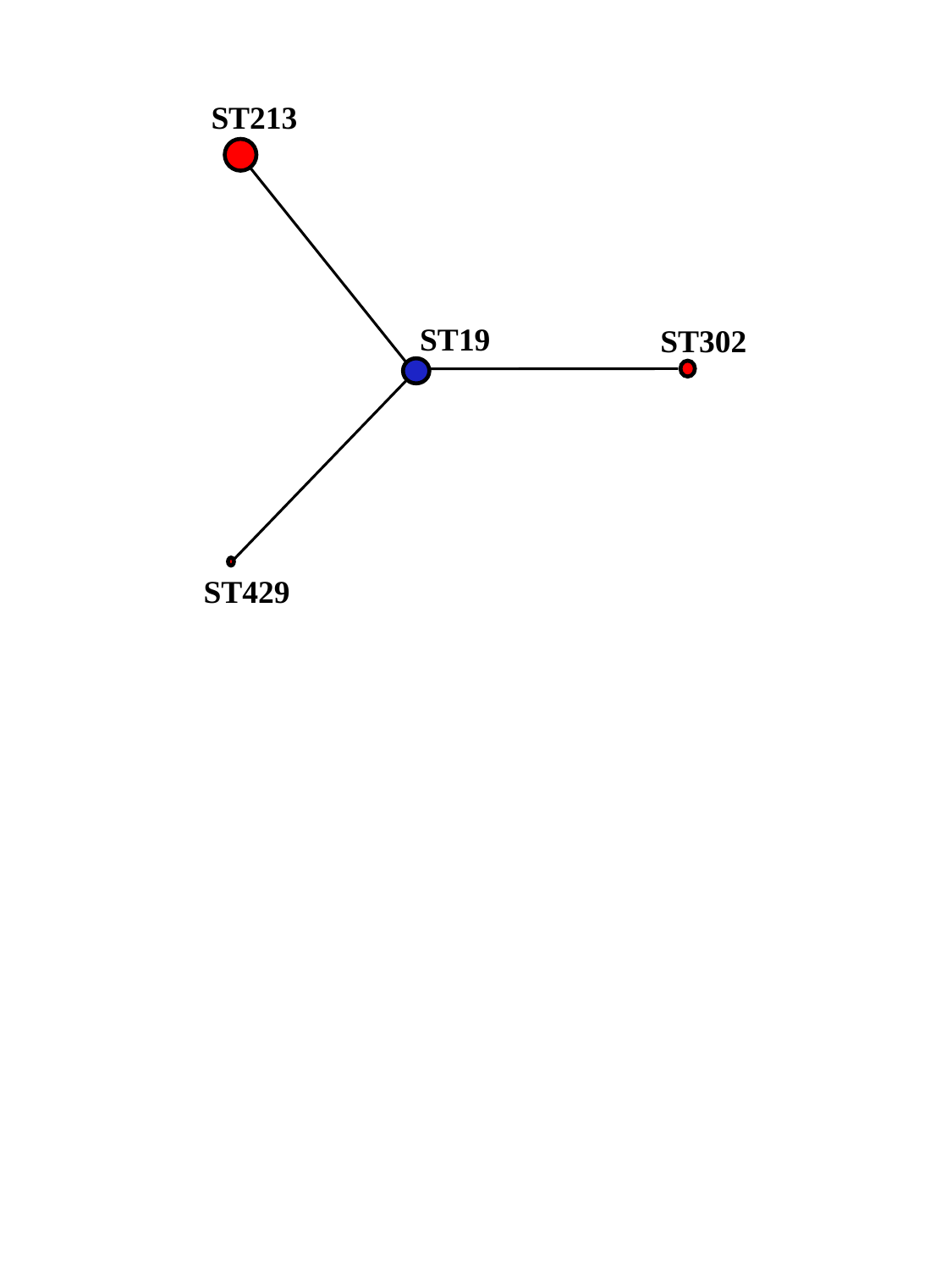

ST213
ST19
ST302
ST429

Supplement: Additional file 1 — Figure S1 – Clonal complex for the four multilocus genotypes found in the Mexican Typhimurium population. Representation of the clonal relatedness of STs. Figure S1 – Clonal complex for the four multilocus genotypes found in the Mexican Typhimurium population. The eBURST diagram show the genetic relationships for 66 Typhimurium strains based on the MLST data. ST 19 was unambiguously (100% bootstrap support) predicted as the founder genotype, with STs 213, 302 and 429 related as single locus variants of ST19. The size of the circles is proportional to the number of isolates belonging to each ST. [file 1471-2180-9-131-S1.ppt]
