# Supplementary material for: Association of virulence plasmid and antibiotic resistance determinants with chromosomal multilocus genotypes in Mexican Salmonella enterica serovar Typhimurium strains
Source: BMC Microbiol. 2009 Jul 3;9:131. doi: 10.1186/1471-2180-9-131 (PMC2715408; doi:10.1186/1471-2180-9-131)
Supplement: Additional file 2 — Table S1 – Complete list of strains and results. The complete list of strains, sampling information and results of the genotypic and phenotypic characterization is presented. Table S1 – Complete list of strains and results. The complete list of strains, sampling information and results of the genotypic and phenotypic characterization is presented. [file 1471-2180-9-131-S2.doc]

|  |  |  |  |  |  | **pSTV** | | |  |  |  |  |  |  |  |  | **Antimicrobial phenotype^a^** | | | | | | | | | | |
| --- | --- | --- | --- | --- | --- | --- | --- | --- | --- | --- | --- | --- | --- | --- | --- | --- | --- | --- | --- | --- | --- | --- | --- | --- | --- | --- | --- |
| **Strain** | **Source** | **State** | **Year** | **ST ^b^** | **PFGE** | ***spvC*** | ***rck*** | ***traT*** | **IP** | ***intI1*** | ***qacEΔ1*** | **SGI1 ^c^** | ***aadA2*** | ***pse1*** | ***tetG*** | ***floR*** | ***AMP*** | ***CHL*** | ***SSS*** | ***STR*** | ***TET*** | **GM** | **KM** | **NAL** | **SXT** | **CRO** | **CIP** |
| **Human source** |  |  |  |  |  |  |  |  |  |  |  |  |  |  |  |  |  |  |  |  |  |  |  |  |  |  |  |
| YUHS 00-68 | Diarrhea | Yucatán | 2000 | 19 | V | - | nt | nt | - | - | - | nt | nt | nt | nt | nt | *S* | *S* | *R* | *R* | *R* | S | S | S | S | 0.12/S- | 0.06/S |
| YUHS 00-87 | Diarrhea | Yucatán | 2000 | 19 | V | - | nt | nt | - | nt | nt | nt | nt | nt | nt | nt | *S* | *S* | *S* | *R* | *R* | S | S | S | S | 0.25/S- | 0.06/S |
| YUHS 00-98 | Diarrhea | Yucatán | 2000 | 19 | V | - | nt | nt | - | nt | nt | nt | nt | nt | nt | nt | *S* | *S* | *R* | *R* | *R* | S | S | R | R | 0.25/S | 0.06/S |
| YUHS 00-141 | Diarrhea | Yucatán | 2000 | 19a | II | - | - | - | SGI | + | + | L-R-RR | + | + | + | + | *R* | *R* | *R* | *R* | *S* | S | S | S | S | 0.06/S- | 0.03/S |
| YUHS 00-204 | Diarrhea | Yucatán | 2000 | 19 | II | + | + | + | SGI | + | + | L-R-RR | + | + | + | + | *R* | *R* | *R* | *R* | *R* | S | S | S | S | 0.06/S- | 0.03/S |
| YUHS 00-228 | Diarrhea | Yucatán | 2000 | 19a | II | + | + | + | SGI | + | + | L-R-RR | + | + | + | + | *R* | *R* | *R* | *R* | *S* | S | S | S | S | 0.25/S | 0.015/S |
| YUHS 01-375 | Diarrhea | Yucatán | 2001 | 19 | III | + | + | + | SGI | + | + | L-R-RR | + | + | + | + | *R* | *R* | *R* | *R* | *R* | S | S | S | R | 0.06/S- | 0.03/S |
| YUHS 01-386 | Diarrhea | Yucatán | 2001 | 19a | III | - | nt | nt | - | nt | nt | nt | nt | nt | nt | nt | *S* | *S* | *S* | *S* | *R* | S | S | S | S | 0.5/S | 0.03/S |
| YUHS 01-392 | Diarrhea | Yucatán | 2001 | 19a | III | + | + | + | - | - | - | nt | nt | nt | nt | - | *S* | *S* | *S* | *R* | *S* | S | S | S | S | 0.25/S- | 0.03/S |
| YUHS 01-400 | Diarrhea | Yucatán | 2001 | 19 | V | - | nt | nt | - | nt | nt | nt | nt | nt | nt | nt | *R* | *S* | *R* | *R* | *R* | S | S | S | R | 0.12/S | 0.03/S |
| YUHS 01-401 | Diarrhea | Yucatán | 2001 | 19 | V | - | nt | nt | - | - | - | nt | nt | nt | nt | w | *S* | *S* | *S* | *R* | *S* | S | S | S | S | 0.12/S- | 0.06/S |
| YUHS 04-82 | Asymptomatic | Yucatán | 2004 | 19 | III | - | - | - | - | - | - | nt | nt | nt | nt | + | *S* | *S* | *R* | *R* | *S* | S | S | S | S | 0.12/S- | 0.03/S |
| YUHS 02-75 | Systemic infection | Yucatán | 2002 | 213 | I | - | nt | nt | 1 | + | - | nt | nt | nt | nt | + | *R* | *R* | *R* | *R* | *R* | R | R | R | R | 64/R+ | 0.06/S |
| YUHS 03-19 | Diarrhea | Yucatán | 2003 | 213 | I | - | nt | nt | 1 | + | - | - | nt | nt | nt | + | *R* | *R* | *R* | *R* | *R* | R | R | R | R | 64/R+ | 1.0/LLR |
| YUHS 03-25 | Asymptomatic | Yucatán | 2003 | 213 | I | - | nt | nt | 1 | + | - | nt | nt | nt | nt | + | *R* | *R* | *R* | *R* | *R* | S | S | S | R | 128/R+ | 0.03/S |
| YUHS 03-26 | Asymptomatic | Yucatán | 2003 | 213 | I | - | nt | nt | 1 | + | - | nt | nt | nt | nt | + | *R* | *R* | *R* | *R* | *R* | S | S | S | R | 32/R+ | 0.03/S |
| YUHS 03-31 | Diarrhea | Yucatán | 2003 | 213 | I | - | nt | nt | 1 | + | - | nt | nt | nt | nt | + | *R* | *R* | *R* | *R* | *R* | R | R | R | R | 64/R+ | 0.12/S |
| YUHS 03-34 | Diarrhea | Yucatán | 2003 | 213 | I | - | nt | nt | 1 | + | - | nt | nt | nt | nt | + | *R* | *R* | *R* | *R* | *R* | R | S | R | R | 64/R+ | 0.12/S |
| YUHS 03-58A | Diarrhea | Yucatán | 2003 | 213 | I | - | nt | nt | 1 | + | - | nt | nt | nt | nt | + | *R* | *R* | *R* | *R* | *R* | R | R | S | R | 32/R | 0.03/S |
| YUHS 03-59 | Diarrhea | Yucatán | 2003 | 213 | I | - | nt | nt | 1 | + | - | nt | nt | nt | nt | + | *R* | *R* | *R* | *R* | *R* | R | R | S | R | 32/R+ | 0.03/S |
| YUHS 03-72 | Diarrhea | Yucatán | 2003 | 213 | I | - | nt | - | - | + | - | nt | nt | nt | nt | + | *S* | *R* | *S* | *R* | *I* | S | S | I | S | 32/R+ | 0.06/S |
| YUHS 03-80 | Diarrhea | Yucatán | 2003 | 213 | I | **+** | **+** | **+** | - | - | - | nt | - | - | nt | + | *R* | *R* | *R* | *R* | *R* | S | S | R | R | 0.25/S- | 0.12/S |
| YUHS 03-84 | Diarrhea | Yucatán | 2003 | 213 | I | - | nt | nt | 1 | + | - | nt | nt | nt | nt | + | *R* | *R* | *R* | *R* | *R* | R | S | R | R | 32/R+ | 0.12/S |
| YUHS 04-15 | Diarrhea | Yucatán | 2004 | 213 | I | - | - | - | - | + | - | nt | nt | nt | nt | + | *R* | *R* | *R* | *R* | *R* | S | S | S | R | 128/R+ | 0.03/S |
| YUHS 04-23 | Asymptomatic | Yucatán | 2004 | 213 | I | - | nt | nt | 1 | + | - | nt | nt | nt | nt | + | *R* | *R* | *R* | *R* | *R* | S | R | R | R | 128/R+ | 0.12/S |
| YUHS 04-31 | Systemic infection | Yucatán | 2004 | 213 | I | - | nt | nt | 1 | + | - | - | + | - | - | + | *R* | *R* | *R* | *R* | *R* | S | S | R | R | 32/R+ | 0.12/S |
| YUHS 04-36 | Diarrhea | Yucatán | 2004 | 213 | I | - | nt | nt | 1 | + | - | nt | nt | nt | nt | + | *R* | *R* | *R* | *R* | *R* | S | S | R | R | 64/R+ | 0.12/S |
| YUHS 04-39 | Diarrhea | Yucatán | 2004 | 213 | I | - | nt | nt | 1 | + | - | nt | nt | nt | nt | + | *R* | *R* | *R* | *R* | *R* | S | S | S | R | 64/R+ | 0.03/S |
| YUHS 04-50 | Systemic infection | Yucatán | 2004 | 213 | I | - | - | nt | - | + | - | nt | nt | nt | nt | + | *R* | *R* | *R* | *R* | *R* | R | R | S | R | 128/R+ | 0.03/S |
| YUHS 04-62 | Diarrhea | Yucatán | 2004 | 213 | I | - | - | - | 1 | + | - | nt | nt | nt | nt | + | *R* | *R* | *R* | *R* | *R* | S | S | S | R | 64/R+ | 0.03/S |
| YUHS 05-26 | Diarrhea | Yucatán | 2005 | 213 | I | - | nt | nt | 1 | + | - | nt | nt | nt | nt | + | *R* | *R* | *R* | *R* | *R* | R | S | R | R | 128/R+ | 0.12/S |
| YUHS 05-75 | Systemic infection | Yucatán | 2005 | 213 | I | - | nt | nt | 1 | + | - | nt | nt | nt | nt | + | *R* | *R* | *R* | *R* | *R* | S | S | R | R | 64/R+ | 0.06/S |
| YUHS 05-78 | Systemic infection | Yucatán | 2005 | 213 | I | - | nt | nt | 1 | w | - | nt | nt | nt | nt | + | *R* | *R* | *R* | *R* | *R* | S | S | R | R | 128/R+ | 0.12/S |
| MIHS 03-14 | Asymptomatic | Michoacán | 2003 | 19 | II | + | + | + | SGI | + | + | L-R-RR | + | + | + | + | *R* | *R* | *R* | *S* | *R* | S | S | S | S | 0.25/S | 0.03/S |
| MIHS 03-18 | Diarrhea | Michoacán | 2003 | 19 | II | **+** | **+** | **+** | SGI | + | + | L-R-RR | + | + | + | + | *R* | *R* | *R* | *R* | *R* | S | S | S | S | 0.25/S | 0.03/S |
| MIHS 03-26-1 | Asymptomatic | Michoacán | 2003 | 19 | II | **+** | **+** | **+** | SGI | + | + | L-R-RR | + | + | + | + | *R* | *R* | *R* | *S* | *R* | S | S | S | S | 0.12/S | 0.03/S |
| MIHS 02-19 | Diarrhea | Michoacán | 2002 | 213 | I | - | - | - | 1 | + | - | - | nt | nt | nt | + | *R* | *R* | *R* | *S* | *R* | R | R | S | R | 32/R+ | 0.06/S |
| MIHS 05-11A | Diarrhea | Michoacán | 2005 | 213 | I | - | nt | - | 1 | + | - | nt | nt | nt | nt | nt | *S* | *R* | *R* | *R* | *R* | R | R | R | R | 0.12/S- | 0.25/LLR |
| SLHS 01-77 | Diarrhea | San Luis Potosí | 2001 | 19a | III | + | + | + | - | nt | nt | nt | nt | nt | nt | nt | *S* | *S* | *R* | *R* | *R* | S | S | S | S | 0.12/S- | 0.06/S |
| SLHS 01-78 | Diarrhea | San Luis Potosí | 2001 | 19a | III | + | + | + | - | nt | nt | nt | nt | nt | nt | nt | *S* | *S* | *R* | *R* | *R* | S | S | S | S | 0.12/S | 0.06/S |
| SLHS 01-79 | Diarrhea | San Luis Potosí | 2001 | 19a | III | + | + | + | - | - | - | nt | nt | nt | nt | nt | *S* | *S* | *R* | *I* | *R* | S | S | S | S | 0.06/S | 0.03/S |
| SLHS 01-80 | Diarrhea | San Luis Potosí | 2001 | 19a | III | + | + | + | - | nt | nt | nt | nt | nt | nt | nt | *S* | *S* | *R* | *R* | *R* | S | S | S | S | 0.25/S | 0.12/S |
| SLHS 02-20 | Diarrhea | San Luis Potosí | 2002 | 19 | IV | **+** | - | **+** | 4 | + | + | - | nt | nt | - | w | *R* | *S* | *R* | *R* | *R* | S | R | S | R | 0.12/S- | 0.06/S |
| SLHS 02-7 | Asymptomatic | San Luis Potosí | 2002 | 19 | II | + | + | + | SGI | + | + | L-R-RR | + | + | + | + | *R* | *R* | *R* | *S* | *R* | S | S | S | S | 0.12/S | 0.03/S |
| SLHS 02-8 | Asymptomatic | San Luis Potosí | 2002 | 19 | III | + | + | + | - | w | - | nt | nt | nt | nt | nt | *S* | *S* | *S* | *S* | *S* | S | S | S | S | 0.06/S | 0.06/S |
| SLHS 02-12 | Asymptomatic | San Luis Potosí | 2002 | 213 | I | - | nt | nt | 1 | + | w | nt | nt | nt | nt | nt | *R* | *R* | *R* | *R* | *R* | R | S | R | R | 0.25/S | 0.12/S |
| SLHS 03-10 | Diarrhea | San Luis Potosí | 2003 | 213 | I | - | nt | nt | 1 | + | - | nt | nt | nt | nt | + | *R* | *R* | *R* | *R* | *R* | R | S | R | R | 32/R+ | 0.06/S |
| SLHS 03-15 | Diarrhea | San Luis Potosí | 2003 | 213 | I | - | nt | nt | 1 | + | + | nt | nt | nt | nt | nt | *R* | *S* | *R* | *R* | *S* | S | S | R | R | 0.12/S- | 0.5/LLR |
| SLHS 03-9 | Diarrhea | San Luis Potosí | 2003 | 213 | I | - | nt | - | 1 | + | w | - | nt | nt | nt | + | *R* | *R* | *R* | *R* | *R* | R | S | R | R | 64/R+ | 0.06/S |
| SLHS 05-7 | Diarrhea | San Luis Potosí | 2005 | 19a | II | **+** | **+** | **+** | SGI | + | + | L-R-RR | + | + | + | + | *R* | *R* | *R* | *R* | *R* | S | S | S | R | 0.06/S- | 0.03/S |
| SOHS 02-2 | Diarrhea | Sonora | 2002 | 19 | IV | **+** | **+** | **+** | - | w | w | nt | nt | nt | nt | nt | *S* | *S* | *S* | *S* | *S* | S | S | S | S | 0.06/S | 0.03/S |
| SOHS 03-17 | Diarrhea | Sonora | 2003 | 19 | II | + | + | + | SGI | + | + | L-R-RR | + | + | + | + | *R* | *R* | *R* | *R* | *R* | S | S | R | S | 0.06/S | 1.0/LLR |
| SOHS 03-52 | Diarrhea | Sonora | 2003 | 19 | IV | + | + | + | - | w | w | nt | nt | nt | nt | nt | *S* | *S* | *R* | *S* | *S* | S | S | S | S | 0.06/S | 0.03/S |
| SOHS 04-17 | Diarrhea | Sonora | 2004 | 19 | II | + | + | + | SGI | + | + | L-R-RR | + | + | + | + | *R* | *R* | *R* | *R* | *R* | S | S | S | S | 0.25/S | 0.06/S |
| SOHS 04-18 | Diarrhea | Sonora | 2004 | 19 | II | + | + | + | SGI | + | + | L-R-RR | + | + | + | + | *R* | *R* | *R* | *R* | *R* | S | S | S | S | 0.25/S | 0.06/S |
| SOHS 04-31 | Diarrhea | Sonora | 2004 | 19 | V | - | nt | nt | 3 | + | + | - | nt | nt | nt | nt | *R* | *S* | *R* | *R* | *R* | S | S | S | S | 0.25/S | 0.06/S |
| SOHS 04-42 | Diarrhea | Sonora | 2004 | 19 | V | - | nt | nt | - | nt | nt | nt | nt | nt | nt | nt | *S* | *S* | *S* | *R* | *S* | S | S | S | S | 0.12/S | 0.03/S |
| SOHS 02-20 | Asymptomatic | Sonora | 2002 | 302 | III | **+** | **+** | **+** | - | w | w | nt | - | - | nt | w | *S* | *S* | *S* | *S* | *S* | S | S | S | S | 0.06/S | 0.03/S |
| SOHS 02-68 | Systemic infection | Sonora | 2002 | 302 | III | + | + | + | - | nt | nt | nt | nt | nt | nt | nt | *S* | *S* | *S* | *R* | *S* | S | S | S | S | 0.06/S | 0.03/S |
| SOHS 03-1 | Asymptomatic | Sonora | 2003 | 302 | III | + | + | + | - | nt | nt | nt | nt | nt | nt | nt | *S* | *S* | *S* | *S* | *S* | S | S | S | S | 0.06/S | 0.03/S |
| SOHS 04-44 | Diarrhea | Sonora | 2004 | 302 | III | + | + | + | - | w | w | nt | nt | nt | nt | w | *S* | *S* | *R* | *S* | *S* | S | S | S | S | 0.06/S- | 0.03/S |
| SOHS 04-19 | Diarrhea | Sonora | 2004 | 213 | I | - | nt | - | - | w | w | nt | nt | nt | nt | + | *S* | *R* | *R* | *R* | *R* | S | S | S | S | 0.25/S | 0.06/S |
| **Animal source** |  |  |  |  |  |  |  |  |  |  |  |  |  |  |  |  |  |  |  |  |  |  |  |  |  |  |  |
| YUPUS 03-15 | Pork meat | Yucatán | 2003 | 213a | I | - | nt | - | 1 | + | w | nt | + | nt | nt | nt | *R* | *R* | *R* | *R* | *R* | S | S | S | R | 64/R+ | 0.03/S |
| YUPUS 03-18 | Pork meat | Yucatán | 2003 | 213 | I | - | - | - | 1 | + | w | - | + | - | - | + | *R* | *R* | *R* | *R* | *R* | S | S | S | R | 64/R+ | 0.03/S |
| YUPUS 03-32-1 | Pork meat | Yucatán | 2003 | 213a | I | - | nt | nt | 1 | + | - | nt | + | nt | nt | nt | *R* | *R* | *R* | *R* | *R* | R | R | R | R | 64/R+ | 0.12/S |
| MIPUS 02-31 | Pork meat | Michoacán | 2002 | 213a | I | - | nt | nt | 1 | + | w | nt | nt | nt | nt | + | *S* | *R* | *R* | *R* | *R* | R | I | I | R | 0.12/S | 0.03/S |
| MIPUS 02-33 | Pork meat | Michoacán | 2002 | 213a | I | - | nt | nt | 1 | + | - | nt | nt | nt | nt | nt | *S* | *R* | *R* | *R* | *R* | R | I | I | R | 0.06/S | 0.12/S |
| MIPUS 02-34 | Pork meat | Michoacán | 2002 | 213a | I | - | nt | nt | 1 | + | - | nt | nt | nt | nt | nt | *S* | *R* | *R* | *R* | *R* | R | S | R | R | 0.06/S | 0.12/S |
| MIPUS 03-27 | Pork meat | Michoacán | 2003 | 213 | I | - | nt | nt | 1 | + | - | - | nt | nt | nt | + | *S* | *R* | *R* | *R* | *R* | R | R | R | R | 0.06/S | 0.12/S |
| MIPUS 03-43-1 | Pork meat | Michoacán | 2003 | 213a | I | - | nt | nt | - | nt | nt | nt | nt | nt | nt | nt | *S* | *R* | *R* | *R* | *R* | R | S | R | R | 0.06/S | 0.12/S |
| MIPUS 04-42 | Pork meat | Michoacán | 2004 | 213a | I | - | nt | - | 2 | + | + | - | nt | nt | nt | w | *S* | *R* | *R* | *R* | *R* | R | R | R | R | 0.06/S | 0.06/S |
| MIPUS 04-9 | Pork meat | Michoacán | 2004 | 213a | I | - | nt | nt | 1 | + | - | nt | nt | nt | nt | nt | *R* | *S* | *R* | *R* | *R* | R | R | R | R | 0.06/S | 0.25/LLR |
| MIRAPUS 04-3-1 | Pork intestine | Michoacán | 2004 | 19 | I | - | nt | nt | 2 | + | + | - | nt | nt | nt | + | *R* | *R* | *R* | *R* | *R* | R | R | R | R | 0.06/S- | 0.06/S |
| MIRAPUS 04-14 | Pork intestine | Michoacán | 2004 | 213a | I | - | nt | nt | 2 | + | + | - | - | - | w | w | *R* | *S* | *R* | *R* | *R* | R | R | R | R | 0.06/S | 0.06/S |
| MIPUS 03-21 | Pork meat | Michoacán | 2003 | 429 | V | - | nt | nt | - | w | - | nt | nt | nt | nt | nt | *S* | *R* | *S* | *S* | *R* | S | S | S | S | 0.12/S | 0.03/S |
| SLPUS 02-84 | Pork meat | San Luis Potosí | 2002 | 19a | II | + | + | + | SGI | + | + | L-R-RR | + | + | + | + | *R* | *R* | *R* | *R* | *R* | S | S | S | S | 0.06/S | 0.06/S |
| SLPUS 03-42 | Pork meat | San Luis Potosí | 2003 | 19a | II | + | + | + | SGI | + | + | L-R-RR | + | + | + | + | *R* | *R* | *R* | *R* | *R* | S | S | S | S | 0.25/S | 0.06/S |
| SLPUS 05-46 | Pork meat | San Luis Potosí | 2005 | 19a | I | - | nt | nt | 2 | + | + | nt | - | - | w | w | *R* | *S* | *R* | *R* | *R* | R | R | R | R | 0.12/S- | 0.06/S |
| SLPUS 03-2 | Pork meat | San Luis Potosí | 2003 | 213a | I | - | nt | nt | 1 | + | + | nt | nt | nt | nt | nt | *R* | *S* | *R* | *R* | *R* | S | S | R | R | 0.06/S- | 0.03/S |
| SLPUS 03-27-1 | Pork meat | San Luis Potosí | 2003 | 213a | I | - | nt | nt | 1 | + | w | nt | nt | nt | nt | + | *R* | *R* | *R* | *R* | *R* | R | R | R | R | 64/R+ | 0.06/S |
| SLPUS 03-29 | Pork meat | San Luis Potosí | 2003 | 213 | I | - | nt | nt | 1 | + | - | nt | nt | nt | nt | + | *R* | *R* | *R* | *R* | *R* | R | S | R | R | 64/R+ | 0.12/S |
| SLRAPUS 04-2 | Pork intestine | San Luis Potosí | 2004 | 213a | I | - | nt | nt | 1 | + | - | nt | nt | nt | nt | nt | *S* | *R* | *R* |  | *R* | R | S | R | R | 0.12/S | 0.12/S |
| SLRAPUS 04-6 | Pork intestine | San Luis Potosí | 2004 | 213a | I | - | nt | - | 1 | + | - | - | + | - | - | + | *R* | *R* | *R* | *R* | *R* | R | R | R | R | 32/R+ | 0.06/S |
| SLRAPUS 05-32 | Pork intestine | San Luis Potosí | 2005 | 213a | I | - | nt | nt | 1 | + | - | nt | nt | nt | nt | + | *S* | *R* | *R* | *R* | *R* | R | R | S | R | 0.06/S | 0.03/S |
| SOPUS 02-4 | Pork meat | Sonora | 2002 | 19 | I | **+** | **+** | **+** | SGI | + | + | L-R-RR | + | + | + | + | *R* | *R* | *R* | *R* | *R* | S | S | S | S | 0.06/S- | 0.016/S |
| SORAPUS 04-14-2 | Pork intestine | Sonora | 2004 | 213a | I | - | nt | nt | - | nt | nt | nt | nt | nt | nt | nt | *S* | *R* | *R* | *R* | *R* | S | S | S | S | 0.25/S | 0.06/S |
| SORAPUS 04-21 | Pork intestine | Sonora | 2004 | 213a | I | - | nt | nt | - | nt | nt | nt | nt | nt | nt | nt | *R* | *R* | *R* | *R* | *R* | R | R | R | S | 0.12/S | 0.12/S |
| SORAPUS 04-22 | Pork intestine | Sonora | 2004 | 213 | I | - | nt | nt | - | w | w | nt | nt | nt | nt | + | *R* | *R* | *R* | *R* | *R* | R | R | R | S | 0.25/S | 0.12/S |
| SORAPUS 04-29 | Pork intestine | Sonora | 2004 | 213a | I | - | nt | nt | - | nt | nt | nt | nt | nt | nt | nt | *S* | *R* | *R* | *R* | *R* | S | S | S | S | 0.25/S | 0.06/S |
| SORAPUS 05-21 | Pork intestine | Sonora | 2005 | 213a | I | - | nt | nt | - | nt | nt | nt | nt | nt | nt | nt | *S* | *R* | *R* | *R* | *R* | S | S | R | S | 0.12/S | 0.5/LLR |
| YURES 03-7 | Beef meat | Yucatán | 2003 | 213 | I | - | nt | - | 1 | + | w | - | nt | nt | nt | + | *R* | *R* | *R* | *R* | *R* | S | S | S | R | 32/R+ | 0.03/S |
| MIRES 02-35 | Beef meat | Michoacán | 2002 | 213a | I | - | nt | nt | 1 | + | - | nt | nt | nt | nt | nt | *S* | *R* | *R* | *R* | *R* | R | S | R | R | 0.12/S | 0.12/S |
| MIRES 02-36 | Beef meat | Michoacán | 2002 | 213a | I | - | nt | nt | 1 | + | w | nt | nt | nt | nt | nt | *S* | *R* | *R* | *R* | *R* | R | R | R | R | 0.06/S | 0.12/S |
| MIRES 03-12-2 | Beef meat | Michoacán | 2003 | 213a | I | - | nt | nt | - | + | - | nt | nt | nt | nt | + | *R* | *R* | *R* | *R* | *R* | S | S | S | S | 32/R+ | 0.03/S |
| MIRES 04-4 | Beef meat | Michoacán | 2004 | 213 | I | - | - | - | 2 | + | + | - | nt | nt | nt | nt | *R* | *S* | *R* | *R* | *R* | R | R | R | R | 0.06/S | 0.06/S |
| SLRES 02-100-2 | Beef meat | San Luis Potosí | 2002 | 19 | II | **+** | **+** | **+** | SGI | + | + | L-R-RR | + | + | + | + | *R* | *R* | *R* | *R* | *R* | S | S | S | S | 0.12/S- | 0.03/S |
| SLRES 02-89 | Beef meat | San Luis Potosí | 2002 | 19a | II | + | + | + | SGI | + | + | L-R-RR | + | + | + | + | *R* | *R* | *R* | *R* | *R* | S | S | S | R | 0.12/S | 0.03/S |
| SLRES 03-40 | Beef meat | San Luis Potosí | 2003 | 19a | IV | **+** | - | **+** | - | - | - | nt | nt | nt | nt | - | *R* | *S* | *S* | *S* | *S* | S | S | S | S | 0.25/S | 0.03/S |
| SLRES 04-23-2 | Beef meat | San Luis Potosí | 2004 | 19a | II | + | + | + | SGI | + | + | L-R-RR | + | + | + | + | *R* | *R* | *R* | *R* | *R* | S | S | S | S | 0.06/S | 0.03/S |
| SLRES 02-108 | Beef meat | San Luis Potosí | 2002 | 213a | I | - | nt | nt | 1 | + | - | - | nt | nt | nt | nt | *S* | *R* | *R* | *R* | *R* | R | S | R | R | 0.12/S | 0.12/S |
| SLRES 03-55-2 | Beef meat | San Luis Potosí | 2003 | 213a | I | - | nt | nt | 1 | + | - | nt | nt | nt | nt | nt | *S* | *R* | *R* | *R* | *R* | R | S | R | R | 0.25/S- | 0.12/S |
| SLRES 05-138 | Beef meat | San Luis Potosí | 2005 | 213a | I | - | nt | nt | 1 | + | - | nt | nt | nt | nt | nt | *S* | *R* | *R* | *R* | *R* | R | R | R | R | 0.06/S- | 0.06/S |
| SLRARES 04-5 | Beef intestine | San Luis Potosí | 2004 | 19a | II | + | + | + | SGI | + | + | L-R-RR | + | + | + | + | *R* | *R* | *R* | *R* | *R* | S | S | S | S | 0.06/S | 0.03/S |
| SLRARES 04-8 | Beef intestine | San Luis Potosí | 2004 | 213 | I | - | - | - | 1 | + | - | - | nt | nt | nt | + | *R* | *R* | *R* | *R* | *R* | R | R | R | R | 32/R+ | 0.06/S |
| SORES 04-45 | Beef meat | Sonora | 2004 | 213 | I | - | - | - | 1 | + | + | - | nt | nt | nt | + | *R* | *R* | *R* | *R* | *R* | S | S | R | R | 0.06/S- | 1.0/LLR |
| SORES 05-2 | Beef meat | Sonora | 2005 | 213a | I | - | nt | nt | - | nt | nt | nt | nt | nt | nt | nt | *S* | *R* | *R* | *R* | *R* | S | S | R | S | 0.06/S | 0.5/LLR |
| SORARES 04-25 | Beef intestine | Sonora | 2004 | 213a | I | - | nt | nt | - | nt | nt | nt | nt | nt | nt | nt | *S* | *R* | *R* | *R* | *R* | S | S | S | S | 0.25/S | 0.06/S |
| SORARES 05-13 | Beef intestine | Sonora | 2005 | 213a | I | - | nt | nt | - | nt | nt | nt | nt | nt | nt | nt | *S* | *R* | *R* | *R* | *R* | S | S | R | S | 0.06/S | 0.5/LLR |
| YUPOLS 03-31 | Chicken meat | Yucatán | 2003 | 213a | I | - | nt | nt | - | w | - | nt | - | - | - | + | *R* | *R* | *R* | *R* | *R* | S | S | S | S | 64/R+ | 0.06/S |
| MIPOLS 04-21 | Chicken meat | Michoacán | 2004 | 19a | I | - | nt | nt | 2 | + | + | nt | - | - | w | w | *R* | *S* | *R* | *R* | *R* | R | R | S | R | 0.06/S | 0.12/S |
| MIPOLS 03-74 | Chicken meat | Michoacán | 2003 | 213a | I | - | - | nt | 1 | + | - | - | nt | nt | nt | + | *R* | *R* | *R* | *R* | *R* | R | S | R | R | 64/R+ | 0.5/LLR |
| MIPOLS 03-75 | Chicken meat | Michoacán | 2003 | 213 | I | - | nt | - | 1 | + | - | nt | nt | nt | nt | + | *R* | *S* | *R* | *R* | *R* | R | S | R | R | 64/R+ | 0.5/LLR |
| SLPOLS 01-134 | Chicken meat | San Luis Potosí | 2001 | 213a | I | - | - | - | 1 | + | - | nt | nt | nt | nt | + | *R* | *R* | *R* | *R* | *R* | R | S | R | R | 32/R+ | 0.03/S |
| SLPOLS 03-4 | Chicken meat | San Luis Potosí | 2003 | 213a | I | - | nt | nt | 1 | + | + | nt | nt | nt | nt | nt | *R* | *S* | *R* | *R* | *S* | S | S | R | R | 0.12/S | 0.5/LLR |

**^a^** The pentaresistance phenotype is highlighted in italics. In the CRO column, the result of PCR *cmy-2* amplifications are denoted by + or – symbols. For CRO and CIP the interpretation of MIC values (see Methods) are provided.

**^b^** The strains for which the three-locus scheme was applied are indicated by a “a” after the ST number.

**^c^** L-R-RR indicates positive results for the amplification of the left, right and right with retronphage junctions of SGI1.

+, - , w and nt symbols denote positive, negative, weak and not tested PCR amplifications products, respectively.

The PCR products for which nucleotide sequences were determined are highlighted with light grey boxes or dark grey boxes for the sequences submitted to GenBank.
