# Supplementary material for: Association of virulence plasmid and antibiotic resistance determinants with chromosomal multilocus genotypes in Mexican Salmonella enterica serovar Typhimurium strains
Source: BMC Microbiol. 2009 Jul 3;9:131. doi: 10.1186/1471-2180-9-131 (PMC2715408; doi:10.1186/1471-2180-9-131)
Supplement: Additional file 3 — Table S2 – Primers used in this study. The primer sequences, amplification sizes, annealing temperatures and references are listed. Table S2 – Primers used in this study. The primer sequences, amplification sizes, annealing temperatures and references are listed. [file 1471-2180-9-131-S3.doc]

**Table S2 - Primers used in this study.**

| Name | Gene | Sequence | Size^a^ bp | T^b^ ^o^C | Reference |
| --- | --- | --- | --- | --- | --- |
| CMY-F | *cmy-2* | ATA ACC ACC CAG TCA CGC | 600 | 58 | [1] |
| CMY-R |  | CAG TAG CGA GAC TGC GCA |  |  |  |
| SpvC-1 | *spvC* | ACT CCT TGC ACA ACC AAA TGC GGA | 550 | 55 | [2] |
| SpvC-2 |  | TGT CTC TGC ATT TCG CCA TCA |  |  |  |
| rck-F | *rck* | TCG TTC TGT CCT CAC TGC | 500 | 50 | [3] |
| rck-B |  | TCA TAG CCC AGA TCG ATG |  |  |  |
| traT-F | *traT* | GAT GGT TAC ACT GGT CAG | 500 | 56 | [3] |
| traT-B |  | TCT GAG ATC TGT ACG TCG |  |  |  |
| CS-F | cassette | GGC ATC CAA GCA GCA AG | variable | 55 | [4] |
| CS-R |  | AAG CAG ACT TGA CCT GA |  |  |  |
| Pse-F | *Pse-1* | GGC AAT CAC ACT CGA TGA TGC GT | 200 | 56 | [5] |
| Pse-R |  | GGC TCA ATA CGG TCT AGA CGA GT |  |  |  |
| STR-F1 | *aadA2* | AGA CGC TCC GCG CTA TAG AAG T | 250 | 58 | [5] |
| STR-R1 |  | CGG ACC TAC CAA GGC AAC GCT |  |  |  |
| U7-L12 | Left | ACA CCT TGA GCA GGG CAA AG | 600 | 60 | [6] |
| LJ-R1 | junction | AGT TCT AAA GGT TCG TAG TCG |  |  |  |
| 104-RJ | Right | TGA CGA GCT GAA GCG AAT TG | 600 | 60 | [6] |
| C9-L2 | junction | AGC AAG TGT GCG TAA TTT GG |  |  |  |
| 104-D | retron | ACC AGG GCA AAA CTA CAC AG | 5,000 | 60 | [6] |
| FloR-F | *floR* | CTT TGG CTA TAC TGG CGA TG | 300 | 58 | [5] |
| FloR-R |  | GAT CAT TAC AAG CGC GAC AG |  |  |  |
| TetG-F | *tetG* | AGC AGC CTC AAC CAT TGC CGA T | 400 | 56 | [5] |
| TetG-R |  | GGT GTT CCA CTG AAA ACG GTC CT |  |  |  |
| Int1-F | *intI1* | GCC TTG CTG TTC TTC TAC GG | 500 | 56 | [7] |
| Int1-R |  | GAT GCC TGC TTG TTC TAC GG |  |  |  |
| qacE1-F | *qacE*Δ*1* | ATC GCA ATA GTT GGC GAA GT | 250 | 56 | [8] |
| qacE1-R |  | CAA GCT TTT GCC CAT GAA GC |  |  |  |

^a^ Approximate product amplification size.

^b^ Annealing temperature.

**References.**

1. M'Zali FH, Heritage J, Gascoyne-Binzi DM, Denton M, Todd NJ, Hawkey PM: **Transcontinental importation into the UK of *Escherichia coli* expressing a plasmid-mediated AmpC-type beta-lactamase exposed during an outbreak of SHV-5 extended-spectrum beta-lactamase in a Leeds hospital.** *J Antimicrob Chemother* 1997, **40:**823-831.

2. Chiu CH, Ou JT: **Rapid identification of *Salmonella* serovars in feces by specific detection of virulence genes, *invA* and *spvC*, by an enrichment broth culture-multiplex PCR combination assay.** *J Clin Microbiol* 1996, **34:**2619-2622.

3. Guerra B, Soto S, Helmuth R, Mendoza MC: **Characterization of a self-transferable plasmid from *Salmonella enterica* serotype typhimurium clinical isolates carrying two integron-borne gene cassettes together with virulence and drug resistance genes.** *Antimicrob Agents Chemother* 2002, **46:**2977-2981.

4. Levesque C, Piche L, Larose C, Roy PH: **PCR mapping of integrons reveals several novel combinations of resistance genes.** *Antimicrob Agents Chemother* 1995, **39:**185-191.

5. Chiu CH, Su LH, Chu CH, Wang MH, Yeh CM, Weill FX, Chu C: **Detection of multidrug-resistant *Salmonella enterica* serovar typhimurium phage types DT102, DT104, and U302 by multiplex PCR.** *J Clin Microbiol* 2006, **44:**2354-2358.

6. Boyd D, Peters GA, Cloeckaert A, Boumedine KS, Chaslus-Dancla E, Imberechts H, Mulvey MR: **Complete nucleotide sequence of a 43-kilobase genomic island associated with the multidrug resistance region of *Salmonella enterica* serovar Typhimurium DT104 and its identification in phage type DT120 and serovar Agona.** *J Bacteriol* 2001, **183:**5725-5732.

7. Ng LK, Mulvey MR, Martin I, Peters GA, Johnson W: **Genetic characterization of antimicrobial resistance in Canadian isolates of *Salmonella* serovar Typhimurium DT104.** *Antimicrob Agents Chemother* 1999, **43:**3018-3021.

8. Sandvang D, Aarestrup FM, Jensen LB: **Characterisation of integrons and antibiotic resistance genes in Danish multiresistant *Salmonella enterica* Typhimurium DT104.** *FEMS Microbiol Lett* 1998, **160:**37-41.
